# Nichtvirale Infektionen der Leber

**DOI:** 10.1007/s00108-024-01678-3

**Published:** 2024-03-14

**Authors:** A. Deibel, B. Müllhaupt

**Affiliations:** Klinik für Gastroenterologie und Hepatologie, Swiss HPB und Transplantationszentrum, Universitätsspital Zürich und Universität Zürich, Rämistrasse 100, 8091 Zürich, Schweiz

**Keywords:** Bakterieller Leberabszess, Brucellose, Aktinomykose, Hepatische Echinokokkose, Fasziolose, Liver abscess, bacterial, Brucellosis, Actinomycosis, Echinococcosis, hepatic, Fascioliasis

## Abstract

Die nichtviralen Infektionen der Leber sind verglichen mit den viralen Entitäten selten bis sehr selten. Sie können durch verschiedenste Bakterien, Helminthen, Protozoen und Pilze verursacht werden, bei denen es oft im Rahmen der Dissemination zu einer Leberbeteiligung kommt. Einige dieser Infektionen betreffen insbesondere immunsupprimierte Personen, andere müssen vor allem nach Reisen in tropische Länder in die differenzialdiagnostischen Überlegungen einbezogen werden. Erfolgt die Infektion durch orale Aufnahme von Eiern, wie etwa bei der zystischen und alveolären Echinokokkose, ist die Leber oft das am häufigsten betroffene Organ. Aufgrund der Vielzahl nichtviraler Leberinfektionen und ihrer unterschiedlichen klinischen Manifestationen ist eine umfassende Diskussion aller potenziellen Erreger und ihrer Auswirkungen im Rahmen dieser Arbeit nicht möglich. Daher können in diesem Kontext nur einige wenige Erkrankungen detaillierter besprochen werden.

## Abstract (en)

Non-viral infections of the liver are rare to very rare compared to viral infections. They can be caused by various bacteria, helminths, protozoa, and fungi, often leading to liver involvement during dissemination. Some of these infections affect in particular immunocompromised individuals, while others need to be considered in the differential diagnostic work-up in patients returning from tropical countries. In cases where the infection occurs through oral ingestion of eggs, such as in cystic and alveolar echinococcosis, the liver is often the most commonly affected organ. Due to the diversity of non-viral liver infections and their varied clinical manifestations, a comprehensive discussion of all potential pathogens and their effects is not within the scope of this article. Therefore, only a few of these conditions will be discussed in more detail.

Nichtvirale Infektionen der Leber sind selten und können durch Bakterien, Parasiten oder Pilze verursacht werden (Tab. 1). Die klinische Präsentation ist sehr variabel und kann sich in einer asymptomatischen Erhöhung der Leberwerte, einer klinisch manifesten Hepatitis, zum Teil mit Ikterus, oder als raumfordernder Prozess (Abszess oder Granulom) manifestieren. Die Beteiligung der Leber im Rahmen einer Sepsis und die bakterielle Cholangitis werden in dieser Übersichtsarbeit nicht diskutiert.

**Table 1:** 

Bakterien	Helminthen	Protozoen	Pilze
Bakterieller Leberabszess*	Zystische Echinokokkose*	Amöbiasis	Hepatosplenische Candidiasis
Brucellose*	Alveoläre Echinokokkose*	Malaria	Histoplasmose
Q‑Fieber*	Fasziolose*	Leishmaniose	*Pneumocystis jirovecii*
Aktinomykose*	Schistosomiasis	Toxoplasmose	Aspergillose
Katzenkratzkrankheit	Strongyloidiasis	Kryptosporidiose	Kryptokokkose
Tuberkulose	Enterobiasis		Mikrosporidiose
Salmonellenhepatitis	Toxocariasis		
Tularämie	Capillariasis		
Melioidose	Clonorchiasis		
Listeriose	Ascariasis		
Syphilishepatitis			
Leptospirose			
Lyme-Borreliose			

*Erkrankungen, die im vorliegenden Beitrag diskutiert werden

## Bakterien

### Bakterieller Leberabszess

Ein Leberabszess entwickelt sich als Folge einer eitrigen Cholangitis, durch eine hämatogene Streuung oder portale Pyämie (Appendizitis, Divertikulitis) und seltener durch eine direkte Inokulation. Verschiedene Studien aus den USA, China und Europa haben gezeigt, dass die jährliche Inzidenz eitriger Leberabszesse in den letzten Jahren zugenommen hat [2]. Der Leberabszess ist vor allem eine Erkrankung des älteren Patienten, so ist die Mehrzahl der Erkrankten zwischen 60 und 80 Jahre alt. Das häufigste Symptom ist Fieber, gefolgt von Abdominalschmerzen und Müdigkeit. Meist handelt es sich um solitäre Abszesse im rechten Leberlappen. Blutkulturen sind in knapp 50 % der Fälle positiv. Am häufigsten finden sich *Streptococcus* spp., *Escherichia* *coli, Klebsiella *spp. oder eine polymikrobielle Abszessflora. Durch Kultivierung von Abszessmaterial kann sogar in bis zu 75 % der Fälle zumindest ein Erreger isoliert werden. Im Gegensatz zu den Blutkulturen lassen sich in Abszesskulturen häufiger mehrere Keime nachweisen, oft auch noch nach Beginn einer antibiotischen Therapie [2].

Bei kleinen Abszessen (3–5 cm) kann eine alleinige antibiotische Behandlung ausreichend sein. Die empirische antibiotische Behandlung sollte intravenös erfolgen und gramnegative Bakterien, grampositive Kokken und Anaerobier abdecken, beispielsweise mit Cephalosporinen der dritten Generation und Metronidazol oder Piperacillin/Tazobactam, die nach Erhalt der Kulturergebnisse resistenzgerecht angepasst werden. Normalerweise werden die Antibiotika etwa 3 Wochen i.v. verabreicht, und die gesamte Therapiedauer sollte 4–6 Wochen betragen [3]. Größere Abszesse müssen oft auch punktiert oder drainiert werden. Gegenüber repetitiven Punktionen soll die Drainage eine höhere Erfolgsrate aufweisen, mit schnellerer klinischer Besserung und Größenabnahme des Abszesses. Bezüglich der Hospitalisationsdauer und der Komplikationsrate unterscheiden sich die beiden Methoden nicht [4].

### Brucellose

Die Brucellose ist mit mehr als 500.000 neuen Fällen pro Jahr die häufigste zoonotische Infektion weltweit [5]. In den mittel- und nordeuropäischen Ländern werden nur sehr wenige Brucellosefälle pro Jahr beobachtet. In anderen Regionen, insbesondere in Zentralasien und in Syrien, hat die Brucellose stark zugenommen. Die Erreger der Gattung *Brucella* sind kleine, fakultativ intrazelluläre gramnegative Kokken. *B. melitensis *(Maltafieber), *B. abortus (Morbus Bang), B. suis* und *B. canis* können den Menschen infizieren. Sie werden durch Kontakt mit Flüssigkeiten infizierter Zuchttiere, insbesondere durch Verzehr nichtpasteurisierter Milchprodukte, oder durch Inhalation von Aerosolpartikeln aufgenommen. Die Brucellose ist eine systemische Erkrankung mit einem breiten klinischen Spektrum. Bis zu 90 % der Infektionen können subklinisch verlaufen. Die akute Infektion präsentiert sich meist als febrile Erkrankung mit

- Nachtschweiß,
- Arthralgien,
- Myalgien,
- Rückenschmerzen,
- Gewichtsverlust und
- Schwäche.

Eine seltene Komplikation der Brucellose ist das hepatische Brucellom

Bei der Untersuchung können eine Hepatosplenomegalie und/oder Lymphadenopathie auffallen. Eine Begleithepatitis ist häufig und manifestiert sich als leichte Transaminitis. In der Leberbiopsie lassen sich oft Granulome nachweisen. Wird die Infektion nicht erkannt oder nicht korrekt behandelt, ist ein chronischer Erkrankungsverlauf (Dauer über ein Jahr) möglich. Die Diagnose wird mittels Kultur (Blutkultur, Knochenmarkskultur), serologischer Untersuchung (Serumagglutinationstest und „enzyme-linked immunosorbent assay“ [ELISA]) sowie Polymerase-Kettenreaktion (PCR) gestellt.

Eine seltene Komplikation der Brucellose ist das hepatische Brucellom, insgesamt sind seit 1904 etwa 60 Fälle publiziert [6]. Die klinischen Zeichen sind unspezifisch. Beobachtet werden unter anderem

- undulierendes Fieber über 1–2 Monate,
- Schüttelfrost,
- Nachtschweiß,
- Malaise,
- Gewichtsverlust und
- Schmerzen im rechten Oberbauch.

**Figure 1:**
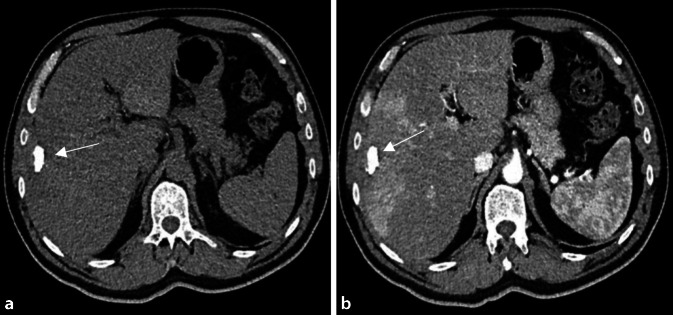

Im Labor finden sich ein erhöhtes C‑reaktives Protein (CRP) und erhöhte Leberwerte, vor allem erhöhte Cholestaseparameter. Die Diagnose beruht auf den üblichen Tests. Allerdings sind die Blut- und Gewebekulturen im Gegensatz zur akuten Brucellose deutlich weniger sensitiv. Entscheidend für die Diagnose sind die radiologischen Veränderungen. In der Computertomographie (CT) erscheint das Brucellom meist als singulärer hypodenser Herd mit einer oder mehreren relativ großen Verkalkungen. Eine zentrale Verkalkung mit umgebender Flüssigkeitskollektion wird als typisch beschrieben (Abb. 1). Für die chronische Brucellose gibt es kein etabliertes Therapieschema. In der Arbeit von Barutta et al. [6] wurde meist Rifampicin (900 mg/Tag; manchmal 1200 mg/Tag oder 20 mg/kg pro Tag) in Kombination mit Doxycyclin 200 mg per os verwendet. Die antibiotische Behandlung allein ist jedoch nur selten (in 20–40 % der Fälle) erfolgreich und muss oft mit perkutanen Drainageverfahren oder chirurgischen Interventionen kombiniert werden.

### Q-Fieber

**Figure 2:**
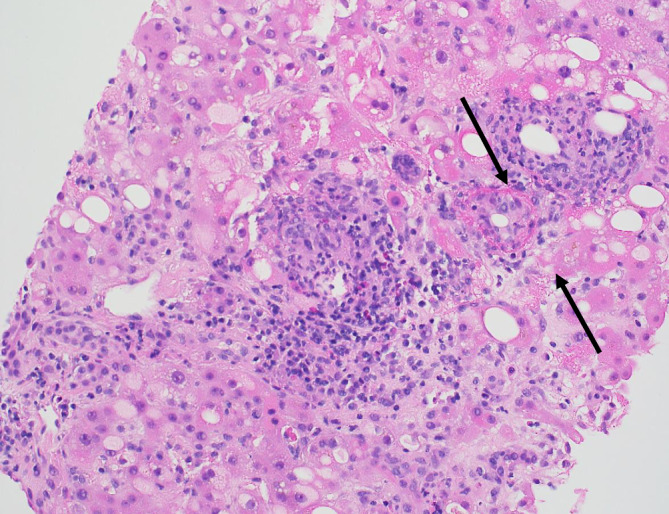

Das Q‑Fieber ist eine weltweit vorkommende Zoonose, die durch *Coxiella burnetii* verursacht wird. Die Übertragung auf den Menschen erfolgt in der Regel durch Inhalation von infizierten Aerosolpartikeln. Die akute Q‑Fieber-Erkrankung verläuft in etwa 60 % der Fälle asymptomatisch, während es bei den restlichen 40 % zu einer selbstlimitierten, grippeähnlichen Erkrankung kommt. Die Diagnose wird entweder serologisch oder auf Grundlage einer positiven PCR gestellt. Die akute, symptomatische Q‑Fieber-Erkrankung wird mit Doxycyclin 100 mg/Tag für 14 Tage behandelt. Eine Hepatitis wird in etwa 40 % der Fälle beobachtet [7]. Histologisch lassen sich meist Granulome nachweisen, wobei das typische Fibrinringgranulom mit einer zentralen Fettvakuole, einem Fibrinring und aktivierten Makrophagen in etwa 50 % der Fälle nachweisbar ist (Abb. 2).

### Aktinomykose der Leber

Die Aktinomykose ist eine seltene, chronisch eitrige, granulomatöse Infektion, die durch nichtsporenbildende, filamentöse, anaerobe grampositive Bakterien des Genus *Actinomyces* verursacht wird. Diese Mikroorganismen sind Teil der normalen Flora des Oropharynx, des Gastrointestinaltrakts und des weiblichen Genitaltrakts. Die Aktinomykose wird je nach Befallsmuster in eine orozervikofaziale (50 % der Fälle), thorakale (15–20 %) oder abdominopelvine Form (etwa 20 %) eingeteilt [8]. Eine Leberbeteiligung ist selten und wird bei etwa 15 % der abdominalen Infektionen beobachtet. Die hepatische Aktinomykose betrifft etwas häufiger immunkompetente Männer mit einem mittleren Alter von 49 Jahren [9]. Häufigste Symptome sind

- Fieber (57,7 %), gefolgt von
- Abdominalschmerzen (52,1 %),
- Gewichtsverlust (45,1 %),
- Anorexie (27,5 %) und
- Schüttelfrost (12,7 %).

**Figure 3:**
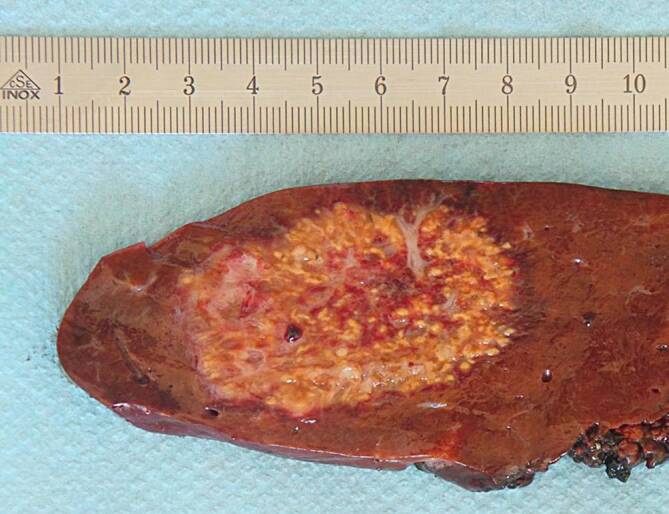

Die Erkrankung zeigt aber oft einen indolenten Verlauf mit einer Symptomdauer von durchschnittlich mehr als 2 Monaten bis zur Diagnose. Bei der klinischen Untersuchung fallen eine Druckdolenz im rechten Oberbauch und eine Hepatomegalie auf. In der Laboruntersuchung finden sich eine Leukozytose sowie eine Erhöhung der Entzündungsparameter und der alkalischen Phosphatase. Radiologisch präsentieren sich die Läsionen entweder als abszessähnliche, zystische Raumforderungen oder als solide tumorverdächtige Knoten. Die Diagnose wird histologisch nach perkutaner Biopsie und Drainage oder nach chirurgischer Resektion gestellt. In der histologischen Untersuchung zeigen sich multiple Abszesse mit akut abszedierender Cholangitis mit Drusen („sulfur granules“). In Spezialfärbungen (Giemsa- und Grocott-Färbung) lassen sich oft typische filamentöse Strukturen nachweisen, vereinbar mit *Actinomyces*-Kolonien (Abb. 3). Als prädisponierender Faktor findet sich am häufigsten

- ein vorangegangener Eingriff im Bauchraum oder im kleinen Becken,
- ein Diabetes mellitus,
- Alkoholmissbrauch,
- Karies oder
- Zahnabszesse.

In der Therapie werden Antibiotika allein oder in Kombination mit einer chirurgischen oder perkutanen Drainage eingesetzt. Die Dauer der Antibiotikatherapie beträgt in der Regel mehrere Monate.

## Helminthen

### Echinokokken

*Echinococcus granulosus *(EG; Hundebandwurm) kann in den meisten Regionen der Welt nachgewiesen werden, am stärksten betroffen sind Regionen in Asien, dem Balkan, Osteuropa, der Türkei und Südamerika. Die Verbreitung von *Echinococcus multilocularis* (EM; Fuchsbandwurm)*, *dem Erreger der alveolären Echinokokkose (AE), ist auf die nördliche Halbkugel beschränkt. EM findet sich vor allem in China (91 % aller Fälle), Zentralasien, Zentraleuropa (französischer Jura, Schweizer Mittelland, Schwäbische Alb) und seit Kürzerem auch in den baltischen Staaten.

### Zystische Echinokokkose

**Figure 4:**
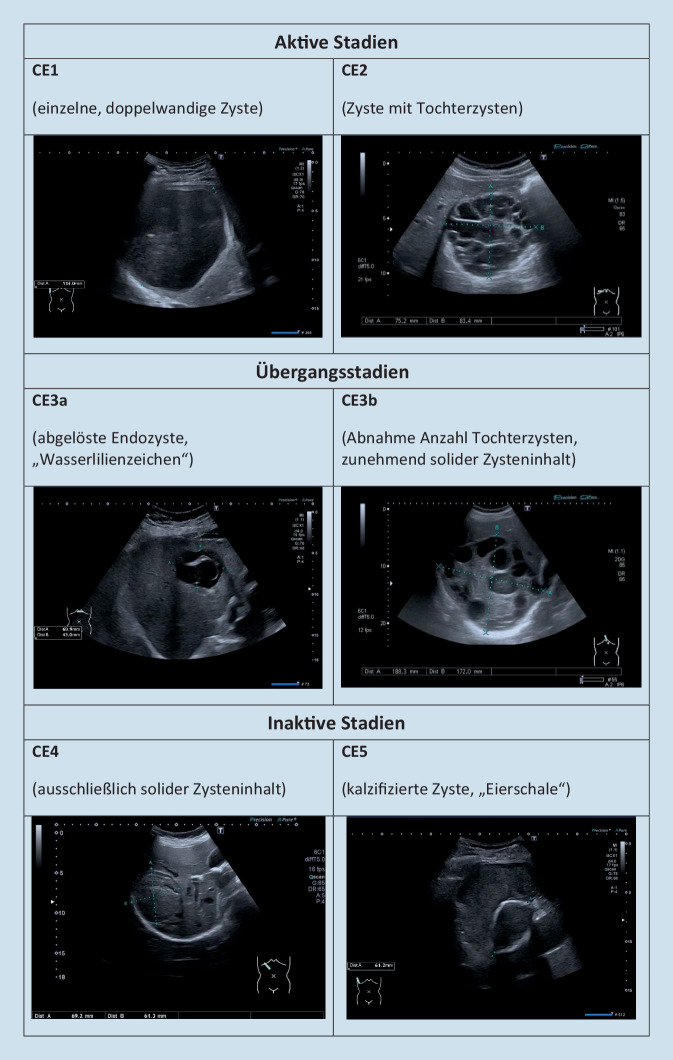

Schlüssel zur Diagnose sind die Herkunft des Patienten aus einem Risikogebiet und der Nachweis von Zysten mit einer sichtbaren Wand in der Leber oder anderen Organen. Bildgebung der Wahl ist der Ultraschall, aber gelegentlich können auch eine CT oder Magnetresonanztomographie indiziert sein. Die Zysten werden gemäß der World Health Organization Informal Working Group on Echinococcosis (WHO-IWGE) auf Grundlage der klassischen Ultraschallpräsentation in verschiedene Stadien eingeteilt, die auch für die Therapiewahl entscheidend sind [10]. CE1 und CE2 gelten als aktive Zysten, CE3 (CE3a und CE3b) als Zysten im Übergangsstadium und CE4 und CE5 als inaktive Zysten (Abb. 4). Die serologische Untersuchung gilt in der Primärdiagnostik als unzuverlässig, kann aber zur Bestätigung eines klinischen Verdachts nützlich sein.

Bei unkomplizierten CE4- oder CE5-Zysten kann eine Watch-and-wait-Strategie verfolgt werden

Zur Behandlung der CE gibt es grundsätzlich vier Optionen:

- Medikamentöse Therapie
- Minimal-invasive perkutane Verfahren
- Chirurgie
- Watch-and-wait-Strategie

Leider gibt es keine randomisierten Studien, die die verschiedenen Optionen vergleichen. Zur medikamentösen Therapie werden die Benzimidazole (BMZ) Albendazol und Mebendazol eingesetzt, die vor allem bei Zysten im Stadium CE1 und CE3a als effektiv gelten. Es ist wichtig, dass BMZ immer mit Fett (Butter, Olivenöl) eingenommen werden, damit therapeutische Blutspiegel erreicht werden. BMZ werden auch immer zusammen mit den perkutanen oder operativen Verfahren eingesetzt. Als minimal-invasives perkutanes Verfahren ist die *p*erkutane Zystenpunktion, *A*spiration des Zysteninhalts, *I*njektion von hypertoner Kochsalzlösung oder Alkohol und *R*easpiration (PAIR) mit ihren Variationen beschrieben [11]. Ziel der Chirurgie sollte die totale Entfernung der Zyste sein, um die Gefahr von lokalen Komplikationen und Rückfällen zu minimieren. Eine partielle Zystektomie hat den Nachteil, dass dabei die Adventitia der Zyste zurückbleibt, was eine Ursache für lokale Komplikationen oder Rezidive sein kann [11]. Schlussendlich können Patienten mit unkomplizierten Zysten im Stadium CE4 oder CE5 auch ohne Therapie überwacht werden. Die Zysten bleiben in der Regel hinsichtlich ihrer Größe stabil oder degenerieren weiter. Voraussetzung für diese Option ist eine regelmäßige Nachkontrolle über mindestens 10 Jahre.

### Alveoläre Echinokokkose

Die Diagnose der AE basiert auf [10]

- den klinischen Befunden (langsam wachsender Lebertumor, als Zufallsbefund oder aufgrund von Beschwerden detektiert),
- den epidemiologischen Gegebenheiten (wohnhaft in Endemiegebiet),
- der Bildgebung,
- der serologischen Untersuchung und
- gelegentlich dem histologischen oder molekularen Nachweis.

**Figure 5:**
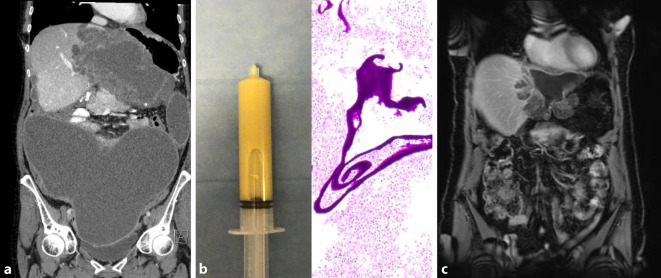

Im Frühstadium kann die AE durch eine chirurgische Resektion und eine 2‑jährige BMZ-Behandlung geheilt werden [11]. Allerdings sind nur etwa 20–50 % der Fälle bei Diagnosestellung operabel. Eine nichtkurative chirurgische Intervention sollte vermieden werden. Bei Inoperabilität ist eine lebenslange medikamentöse Therapie mit BMZ empfohlen [10]. Neuere Daten weisen jedoch darauf hin, dass bei bis zu einem Drittel dieser Patienten im Verlauf (nach mindestens 2‑jähriger BMZ-Therapie) eine Therapiepause möglich ist [12, 13]. Diese Fälle lassen sich mit einer Kombination aus negativem Befund des serologischen Markers Anti-Em18 und negativer 1 h-Fluordesoxyglukose-Positronenemissionstomographie (1 h-FDG-PET) oder negativem Anti-Em2^plus^ und negativer 3 h-FDG-PET identifizieren [12, 13]. Die Lebertransplantation sollte nur in ausgewählten Fällen als Therapieoption evaluiert werden [10]. Insgesamt ist die Prognose der Patienten mit AE heute gut (Abb. 5; [14]).

### Fasziolose

**Figure 6:**
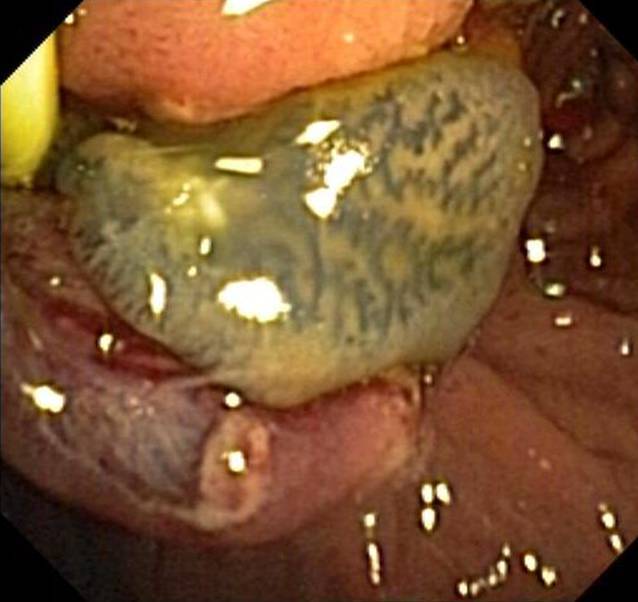

Die Fasziolose ist die häufigste Trematodeninfektion weltweit. *Fasciola* *hepatica* und *F. gigantica* kommen bei Rindern, Schafen und anderen Pflanzenfressern vor. Die adulten Würmer bewohnen die großen Gallenwege des Endwirts, wo sie Eier produzieren, die schlussendlich mit dem Stuhl ausgeschieden werden. Kommt es zur Infektion des Menschen, durchwandern die Larven die Darmwand und dringen durch die Glisson-Kapsel in die Leber ein. Sie wandern als junge Parasiten durch die Leber (akute Infektionsphase), um schließlich in die Gallengänge zu gelangen (chronische Phase), wo sie als erwachsene Würmer beginnen Eier auszuscheiden. Die Symptome beim Menschen sind in der Regel milder Natur und viele Infektionen verlaufen asymptomatisch. Beschwerden beginnen in der Regel 2 Monate nach Ingestion der Metazerkarien (Salat, Wasserkressen) und 1–2 Monate, bevor Eier ausgeschieden werden [15]. Bei der Fasziolose kann eine akute oder Leberphase von einer chronischen oder biliären Phase unterschieden werden. In der akuten Phase können Oberbauchschmerzen, Fieber, Hepatomegalie und eine deutliche Eosinophilie auftreten. Die biliäre oder chronische Phase verläuft oft asymptomatisch. Die adulten Würmer können jedoch einzelne Gallenwege teilweise oder ganz verschließen, was zu rezidivierenden Gallengangskoliken sowie Gallengangsentzündungen führen und intermittierend einen Verschlussikterus hervorrufen kann (Abb. 6).

Die Diagnose wird auf Grundlage des Nachweises der charakteristischen Eier in Stuhl, seltener in Duodenal- und/oder Galleaspirat gestellt. Es ist wichtig, mehrere Stuhlproben einzusenden. Da die Eier erst im chronischen Stadium ausgeschieden werden, ist die Stuhldiagnostik in den Frühstadien unzuverlässig. Hier hilft ein sehr sensitiver und spezifischer ELISA. Eine unspezifische Laborveränderung ist die periphere Eosinophilie, manchmal zusammen mit einer Anämie und mit abnormen Leberfunktionstests.

In den Frühstadien der Fasziolose ist die Stuhldiagnostik unzuverlässig

Medikament der Wahl ist Triclabendazol, das sehr wirksam ist, in der Schweiz und Deutschland aber keine Zulassung hat. Seit Februar 2019 ist das Medikament von der US Food and Drug Administration (FDA) für die Behandlung der Fasziolose bei Menschen zugelassen. Triclabendazol wird in einer Dosierung von 10 bis 12 mg/kgKG für 1 oder 2 Tage gegeben und ist gut verträglich.

## Fazit für die Praxis

- Die Häufigkeit von Leberabszessen nimmt zu. Blutkulturen und Abszesspunktion sind die wichtigsten diagnostischen Schritte. An diese schließt sich eine resistenzgerechte antibiotische Behandlung mit oder ohne Drainage bzw. Punktion an.
- Das hepatische Brucellom ist eine seltene Komplikation der Brucellose. Typische radiologische Veränderungen sind eine oder mehrere große, grobschollige Verkalkungen.
- Die Leberbiopsie zeigt bei der Q‑Fieber-Infektion häufig Granulome, aber das typische Fibrinringgranulom ist nicht immer nachweisbar.
- Die Diagnose der hepatischen Aktinomykose ist schwierig, da der Krankheitsverlauf oft indolent und die klinische Präsentation unspezifisch ist.
- Die zystische Echinokokkose kommt in Mitteleuropa nur bei Patienten vor, die aus Risikogebieten immigriert sind.
- Bei Patienten aus einem Risikogebiet sollte die alveoläre Echinokokkose in Fällen mit Lebertumoren in die differenzialdiagnostischen Überlegungen einbezogen werden.
- Infektionen mit *Fasciola* spp. sind in unseren Regionen selten. Allerdings sind immer lokale Ausbrüche möglich. Klinisch wird zwischen einer hepatischen (akuten) und biliären (chronischen) Phase unterschieden.
